# Establishment and characterization of a novel ovarian high-grade serous carcinoma cell line—IPO43

**DOI:** 10.1186/s12935-022-02600-3

**Published:** 2022-04-30

**Authors:** Fernanda Silva, Filipa Coelho, Ana Peixoto, Pedro Pinto, Carmo Martins, Ann-Sophie Frombach, Vítor E. Santo, Catarina Brito, António Guimarães, Ana Félix

**Affiliations:** 1 Chronic Diseases Research Center, (CEDOC-FCM-UNL), NOVA Medical School, NMS, Universidade NOVA de Lisboa, 1169-056 Lisbon, Portugal; 2 Molecular Pathobiology Research Unit, Portuguese Institute of Oncology Francisco Gentil Lisbon (IPOLFG), 1099-023 Lisbon, Portugal; 3 Department of Genetics, Portuguese Oncology Institute of Porto, Porto, Portugal; 4 IPO Research Center, Portuguese Oncology Institute of Porto, Porto, Portugal; 5 IBET, Instituto de Biologia Experimental E Tecnológica PT, 2781-901 Oeiras, Portugal; 6 Instituto de Tecnologia Química E Biológica António Xavier, Universidade NOVA de Lisboa, 2780-157 Oeiras, Portugal; 7 Medical Oncology Service, IPOLFG, 1099-023 Lisbon, Portugal; 8 Department of Pathology, IPOLFG, 1099-023 Lisbon, Portugal

**Keywords:** Ovarian cancer, Cell lines, Chemoresistance, 3D models

## Abstract

**Background:**

Epithelial ovarian cancer (EOC) is an aggressive and lethal malignancy and novel EOC cell lines with detailed characterization are needed, to provide researchers with diverse helpful resources to study EOC biological processes and cancer experimental therapies.

**Methods:**

The IPO43 cell line was established from the ascitic fluid of a patient with a diagnosis of high-grade serous carcinoma (HGSC) of the ovary, previously treated with chemotherapy.

Cell immortalization was achieved in 2D cell culture and growth obtained in 2D and 3D cell cultures. The characterization of immortalized cells was done by immunocytochemistry, flow cytometry, cell proliferation, chromosomal Comparative Genomic Hybridization (cCGH), STR profile and Next Generation Sequencing (NGS).

**Results:**

Characterization studies confirmed that IPO43 cell line is of EOC origin and maintains morphological and molecular features of the primary tumor. cCGH analysis showed a complex profile with gains and losses of specific DNA regions in both primary ascitic fluid and cell line IPO43. The cell line was successfully grown in a 3D system which allows its future application in more complex assays than those performed in 2D models. IPO43 cell line is resistant to standard drug treatment in vitro.

**Conclusions:**

IPO43 is available for public research and we hope it can contribute to enrich the in vitro models addressing EOC heterogeneity, being useful to investigate EOC and to develop new therapeutic modalities.

## Highlights

- IPOLFG-SOC43 cell line represents the heterogeneity of Epithelial Ovarian Cancer
- Genetic alterations in cancer cells confer a selective advantage
- 3D cultures preserve the phenotypical features of the original tumor

## Introduction

Cell lines provide a powerful model for medical research and in vitro models are an invaluable tool to study the molecular mechanisms and pathways underlying chemoresistance and cancer recurrence [1, 2].

As the development for precision medicine needs to account for tumor and microenvironment heterogeneity, the precise characterization of in vitro model [3], representing different types of ovarian cancer, is essential for accurate preclinical testing. There are at least 100 publicly available ovarian cancer cell lines but their origin and molecular characteristics are not always known. However, efforts have been made to characterize them molecularly [4, 5].

In this study, we aim to describe the characteristics of a new immortalized human cell line derived from a residual high-grade serous carcinoma (HGSC) submitted to neoadjuvant chemotherapy, focusing on cell culture conditions (two-dimensional (2D) monolayer culture and in three-dimensional (3D) spheroid culture), cell proliferation, expression profile of biomarkers and the drug resistance.

The standard treatment for advanced ovarian cancer is cytoreductive surgery followed by platinum-based chemotherapy [6]. Currently, carboplatin and paclitaxel combination is widely used to treat advanced ovarian cancer globally [7]. Even there has been a significant improvement in the clinical response (CR) and overall survival (OS), a large proportion of the patients relapse due to chemoresistance [7, 8].

## Material and methods

### Establishment of a primary cell line of ovarian carcinoma and culture conditions

A successful primary cell culture was established from the ascitic fluid obtained from a caucasian patient diagnosed with ovarian high-grade serous carcinoma, at Instituto Português de Oncologia Francisco Gentil de Lisboa (IPOLFG). The ascitic fluid was collected for therapeutic reasons (9 months after initial diagnosis) and used to establish a cell culture, under patient’s formal consent, accordingly with the institutional Ethical Committee (UIC-772). Clinical details were record and sample was assigned with a reference number to retained anonymity.

Ascites-derived cells were isolated by centrifugation (1200 rpm, 2 min), collected and suspended in culture medium, Dulbecco’s Modified Eagle Medium (catalog no. 41965-039, Invitrogen™ Life Technologies), supplemented with 1% Antibiotic–Antimycotic (catalog no. 15240062, Invitrogen™ Life Technologies) and 10% FBS (fetal bovine serum, catalog no. S0615, Invitrogen™ Life Technologies) and placed into culture, at 37 °C, in an incubator with humidified atmosphere containing 5% CO_2_.

Immortalization occurred spontaneously after six months in continuous culture. The established cell line is maintained in 2D cultures for more than 120 passages.

This cell line was named IPO43 (IPOLFG- Serous Ovarian Carcinoma 43).

The ability of cells to survive and proliferate in low serum conditions was assessed by plating cells in T25 cm^2^ flasks in DMEM supplemented with 1% FBS and cultured for 20 days. The medium was changed every three days (experiments were performed in duplicate).

### Immunohistochemistry

Characterization of the primary ovarian tumor was performed on formalin fixed paraffin embedded tissue (FFPE). Immunostains were done, after antigen retrieval using citrate buffer (0.01 M citric acid adjusted to pH 6.0), with primary antibodies (see Table 1) for 60 min at room temperature (RT). Secondary antibody Dako Real™, Envision™/HRP (ChemMate Envision Kit, K5007; DAKO, USA) was used and stain developed using Peroxidase/DAB. Sections were counterstained with Mayer's hematoxylin. Selected tissues for each antibody were used as positive control and no primary antibodies were used in the negative controls.

**Table 1:** Antigens evaluated and antibodies used to characterize IPO43 cell line

Antibody, clone catalog number, source	Antigen	Function/location	Dilution	Positive control
Anti-Cytokeratin clone AE1/AE3, ISO53, Dako	Cytokeratin type I and II	Cytoskeleton intermediate filament	1:100	Skin
Anti-β-catenin, sc-7199, Santa Cruz Biotechnology	β-catenin	Transcription factor and E-cadherin interacting protein	1:1000	Colon cancer
Anti-CA125 clone OC125, 325 M, Cell Marque	Mucin16 (MUC16) (Cancer antigen 125)	Glycoprotein	1:150	Ovarian cancer
Anti-cytokeratin clone Cam 5.2, 349205, BD Biosciences	Cytokeratin type II	Cytoskeleton	1:100	Appendix
Anti-Calretinin clone DAK-Calret1, M7245, Dako	Calretinin	Calcium-binding protein	Predilute	Mesothelioma
Anti-CK18 conjugated 488 F4772, Sigma-Aldrich	Cytokeratin type I	Cytoskeleton	1:100	Appendix
Anti-Collagen IV Ab6586, Abcam	Alpha 1 and 2 chains type IV collagen	Basement membrane	1:10	Appendix
Anti-E-cadherin 610181, BD Biosciences	E-cadherin	Adherens junction protein	1:80	Breast cancer
Anti-F-actin, 488, Thermo Fisher	Actin in the filamentous form	Cytoskeleton	1:100	Appendix
Anti-HNF1β HPA002083, Sigma-Aldrich	Hepatocyte nuclear factor 1β (HNF1β)	Transcription factor	1:1000	Kidney
Anti-Ki-67 ab16667, Abcam	Nuclear protein in G1, S, M and G2 phase cell cycle	Cell proliferation	1:150	Tonsil
Anti-CINtec p16, clone E6H4, 725-4713, Ventana Medical System	p16INK4a protein	Transcription factor cell cycle regulator	Predilute	Cervical cancer
Anti-p53 clone DO7, 453 M, Cell Marque	p53	Transcription factor cell cycle regulator	1:150	Colon cancer
Anti-Vimentin, clone 3B4, M7020 Dako and V6389, Sigma Aldrich	Vimentin	Cytoskeleton, intermediate filament type III	1:150	Appendix
Anti-PAX8, clone MRQ-50, 760-4618, Ventana Medical System	PAX8	Transcription factor	Predilute	Fallopian tube
Anti-WT1, clone 6F-H2, M3561, Dako	Wilm’s tumor 1 factor	Transcription factor	1:200	Ovarian cancer

### Immunocytochemistry (ICC) and immunofluorescence

Stains on the original ascitic fluid cells preserved in a cell block and in the established cell line were also obtained using the antibodies listed in Table 1.

IPO43 cells, in the exponential growth phase were removed, cytospins prepared and the cell suspension fixed in ice-cold methanol at 4 °C for 30 min, placed in polyethyleneglycol (PEG, P5402, Sigma-Aldrich) for 30 min and stored at RT. Before ICC staining, PEG was removed by soaking in alcohol. Samples were incubated for 1 h with primary antibodies (Table 1). Images were captured in Digital Microimaging Device (version 1.5, Leica Microsystems) in Olympus Bx50F microscope.

For immunofluorescence of the 2D cultures, permeabilization was performed with 0.1% Triton X-100 for 10 min and blocked for 30 min with 0.2% FSG (fish skin gelatin). Primary antibody was incubated for 2 h at RT. Secondary antibody was diluted in 0.125% FSG and incubated for 1 h at RT. Sections were mounted with ProLong containing DAPI (Life Technologies). Samples were visualized using a fluorescence microscope (DMI6000 Leica Microsystems GmBH, Wetzlar, Germany).

For 3D cultures of IPO43 cells, aggregates in suspension were dehydrated using 30% sucrose solution overnight. Samples were transferred in Tissue Teck OCT™ (Sakura Finetek Europe B.V., Zoeterwoude, Netherlands) and stored at -80 °C. Immunofluorescence staining was performed in 10 µm sections, and visualized as indicated above.

### Flow cytometry

Single cell suspension with 1 × 10^6^ cells in 100 μl of ice cold PBS/BSA 0,1% (Bovine serum albumin, Sigma) was obtained and incubated with antibodies (Table 1). Acquisition was performed in a FACScalibur (Becton Dickinson). Data were analyzed with FlowJo (http://flowjo.com/ version 1.44) software.

### Proliferation curves

Cells obtained from 2D cultures were plated at a cell density of 1 × 10^4^ cells/well in a 12-well plate, in DMEM medium supplemented with 10% FBS and 1% antibiotic–antimycotic. Viable cells count was determined after incubation with a solution of 0,1%(v/v) of Trypan Blue dye (Gibco, Invitrogen Corporation, Paisley, UK) at regular time intervals (0 until 9 days). The principle is that dead cells have a damaged cell membrane, internalize the dye and become blue. Both dead and living cells were counted using a Burker Chamber.

Counting was done in three different experiments. Proliferation curves and doubling time was calculated according to the formula:

$${\text{doubling time = }}\frac{{{\text{duration}}*{\text{ log }}\left( {2} \right)}}{{ {\text{Log }}\left( {\text{final concentration}} \right) - {\text{log }}\left( {\text{initial concentration}} \right)}}$$

Cell number and nuclei density were determined by crystal violet method and Fuchs-Rosenthal hemocytometer with a microscope with phase contrast (DM IRB, Leica, Germany).

Proliferation curves were also used to analyze the dynamics of IPO43 cell line to the standard ovarian chemotherapy. Cells (1 × 10^5^ cells/well) were seeded in 24-well and were synchronized under starvation (culture medium without FBS) for eight hours at 37 °C and 5% CO_2_ plates. Cells were exposed to Carboplatin 25 μg/ml, Paclitaxel 10 μg/ml or both drugs. Cells were collected after 24, 48 and 72 h.

### Wound healing assay

Cells were plated onto 12-well plates in DMEM medium supplemented of 10% FBS, reached confluent monolayer and then treated with 5 µg/mL Mitomycin-C (Sigma), an antiproliferative agent, for 3 h at 37 °C in 5% CO_2_. The monolayer was then scratched with a pipette tip. Detached cells were removed with PBS. Images of closure of the wound were captured at 0, 24, 48 and 72 h.

### Chromosomal comparative genomic hybridization (cCGH)

Genomic DNA isolated from the ascitic fluid and derived cell line was done using DNA Purification Protocol for 1–2 Million Cells (Citogene® DNA isolation kit) according to manufacturer’s protocol. cCGH technique was carried out according the method described [9, 10].

For image analysis a Zeiss epifluorescence microscope linked to a Cytovision CGH software program (Cytovision version 7.4, Leica Biosystems, Richmond, USA) was used. At least, 15 metaphases were acquired for the establishment of the cCGH profile. The green (test DNA) to red (reference DNA) fluorescence ratio along the length of the chromosomes was calculated. This high resolution cCGH version uses dynamic standard reference intervals based in the systematic variation seen in normal samples. When the dynamic standard reference intervals do not overlap the confidence limits, the software interprets as either a loss (red) or a gain (green) in the test DNA. Heterochromatic regions in chromosomes 1, 9, 16 and Y, and the p arms of the acrocentric chromosomes were discarded from the analysis.

### Short tandem repeat (STR)

STR Profile was perfomed by the Service of Cell Line Authentication from ATCC, using seventeen short tandem repeat loci plus the gender determining locus, Amelogenin, that were amplified using the commercially PowerPlex® 18D Kit from Promega according to the manufacturer´s protocol. The cell line was processed using the ABI Prism® 3500xl Genetic Analyzer. Date were analyzed using GeneMapper®ID-X v1.2 software (Applied Biosystems). Appropriate positive and negative controls were run and confirmed for the cell line.

### Next generation sequencing (NGS)

Next-generation sequencing (NGS) was performed using the TruSight Hereditary Cancer (Illumina Inc., San Diego, CA, USA) panel, targeting the full coding regions of 113 genes involved in hereditary predisposition to cancer, with the Nextera Flex for Enrichment (Illumina Inc.) library preparation kit, according to the manufacturer’s protocol. Sequencing was carried out in the NextSeq 550 (Illumina Inc.) platform in 2 × 150 bp paired-end runs. Alignment and variant calling were performed using the software NextGENe (SoftGenetics, LLC, State College, PA, USA), with.vcf files being imported into Geneticist Assistant (SoftGenetics, LLC) for variant annotation and filtering. Variants in the *BRCA1*, *BRCA2* and *TP53* were annotated, excluding intronic variants more than 12 bp away from exon–intron boundaries and variants with a variant allele frequency (VAF) < 5%.

### Polymerase chain reaction and Sanger sequencing

The mutational status by extracting genomic DNA using DNA Purification Protocol for 1–2 Million Cells (Citogene®DNA isolation kit) from cell line, according to manufacturer’s protocol. Mutational analysis of *TP53* mutation was determined by polymerase chain reaction (PCR) amplified products encompassing from exons 4, 5, 6, 7, 8 and 9 (Table 2) of the *TP53* gene (which arbor mutational hotspots).

**Table 2:** Primers for PCR of the *TP53* gene (Exons 4–9)

Exon	Primer	Oligonucleotide sequence
4	forward	gacctggtcctctgactgct
4	reverse	caggcattgaagtctcatgg
5	forward	cgtgttccagttgctttatctg
5	reverse	gccagacctaagagcaatcagt
6	forward	ctcagatagcgatggtgagcag
6	reverse	cttaacccctcctcccagagac
7	forward	cctcatcttgggcctgtgtta
7	reverse	tggaagaaatcggtaagaggtg
8–9	forward	ccttactgcctcttgcttctc
8–9	reverse	cattttgagtgttagactggaaactt

The amplified PCR products were analyzed by electrophoresis, on a 2% (w/v) SEA KEM ® GTG ® agarose (50,074, FMC BioProducts) gel (in TBE buffer 1x, diluted from TBE buffer 10 x, EC-860, National diagnostics) labelled with 0.05% (v/v) ethidium bromide 10 mg/ml (15,585–011, Invitrogen), under UV (BioDocAnalyse Transilluminator, Biometra). DNA fragments with expected size were purified using Exonuclease I (20 U/µL) (catalog no. EN0582, ThermoFisher)/FastAP Thermo sensitive Alkaline Phosphatase (1 U/µL) (EF0651, ThermoFisher), according to the manufacturer’s protocol.

Automated sequencing reacting products were purified with Illustra AutoSeq G-50 GFX (27-5340-03, GE Healthcare) according to manufacturer´s protocol. Sequencing reaction was carried out in a T3000 thermocycler (Biometra), purified with AutoSeq G-50 dye terminator removal kit (27–5340-02, GE Healthcare Life Sciences), according to manufacturer’s instructions and analyzed in an ABI Prism™ 3130 Genetic Analyzer (Applied Biosystems).

### Generation of 3D tumor cell spheroids in stirred-tank culture systems

Single cell suspensions of IPO43 cells (0.2 × 10^6^ cell/mL) were inoculated in 125 mL of culture media in stirred-tank vessels with flat centered cap and angled side arms (Corning® Life Sciences) and cultured on a magnetic stirrer, at 37 °C with humidified atmosphere containing 5% CO_2_. Stirring rates of either 40 rpm or 60 rpm were tested. The culture media was the same used in the 2D cultures or supplemented with 10 µM of ROCK (Rho-associated coiled coil forming protein) inhibitor (highly potent, cell-permeable, and selective for ROCK I and II). Cultures were maintained for, at least, 72 h.

### Cell membrane integrity assay

Cell viability was evaluated using fluorescein diacetate (FDA; Sigma–Aldrich, Steinheim, Germany), an intracellular esterase substrate, at 10 μg/mL in PBS to label live cells, and TO-PRO-3 iodide (Invitrogen) at 1 μM in PBS to identify dead cells. Cultures were incubated for 5 min at RT with the fluorescent probes and then analyzed using a fluorescence microscope (DMI6000 Leica Microsystems GmBH, Wetzlar, Germany).

### Spheroid morphometry

Ferret Diameter and the area of the aggregates were determined adjusting the threshold and applying the respective algorithm of FIJI open source software (Rasband, WS, ImageJ, U. S. National Institutes of Health, Bethesda, MD, USA, http://imagej.nih.gov/ij/, 1997–2012) [11] using the same fluorescence microscope.

### PicoGreen assay

Total cell quantification of DNA in the 3D cultures using Quant-iT™ PicoGreen® dsDNA Assay Kit (Life Technologies) according to manufacturer’s protocol.

## Results

### History of the patient from whom IPO43 was derived

The successful primary cell culture was established from the ascitic fluid obtained from a 73-year-old caucasian female patient, diagnosed with an ovarian high grade serous carcinoma, stage IIB (FIGO) with irressectable disease, accordingly to imaging studies at Instituto Português de Oncologia Francisco Gentil de Lisboa. The patient underwent neoadjuvant standard chemotherapy (3 cycles of carboplatin and paclitaxel), followed by surgery and was then submitted to 3 more cycles. In the follow-up, 3 months after finishing chemotherapy, ascitic fluid was collected for therapeutic reasons and for cell culture, under patient’s formal consent.

### Establishment of the characteristics of ascitic cells

**Fig. 1:**
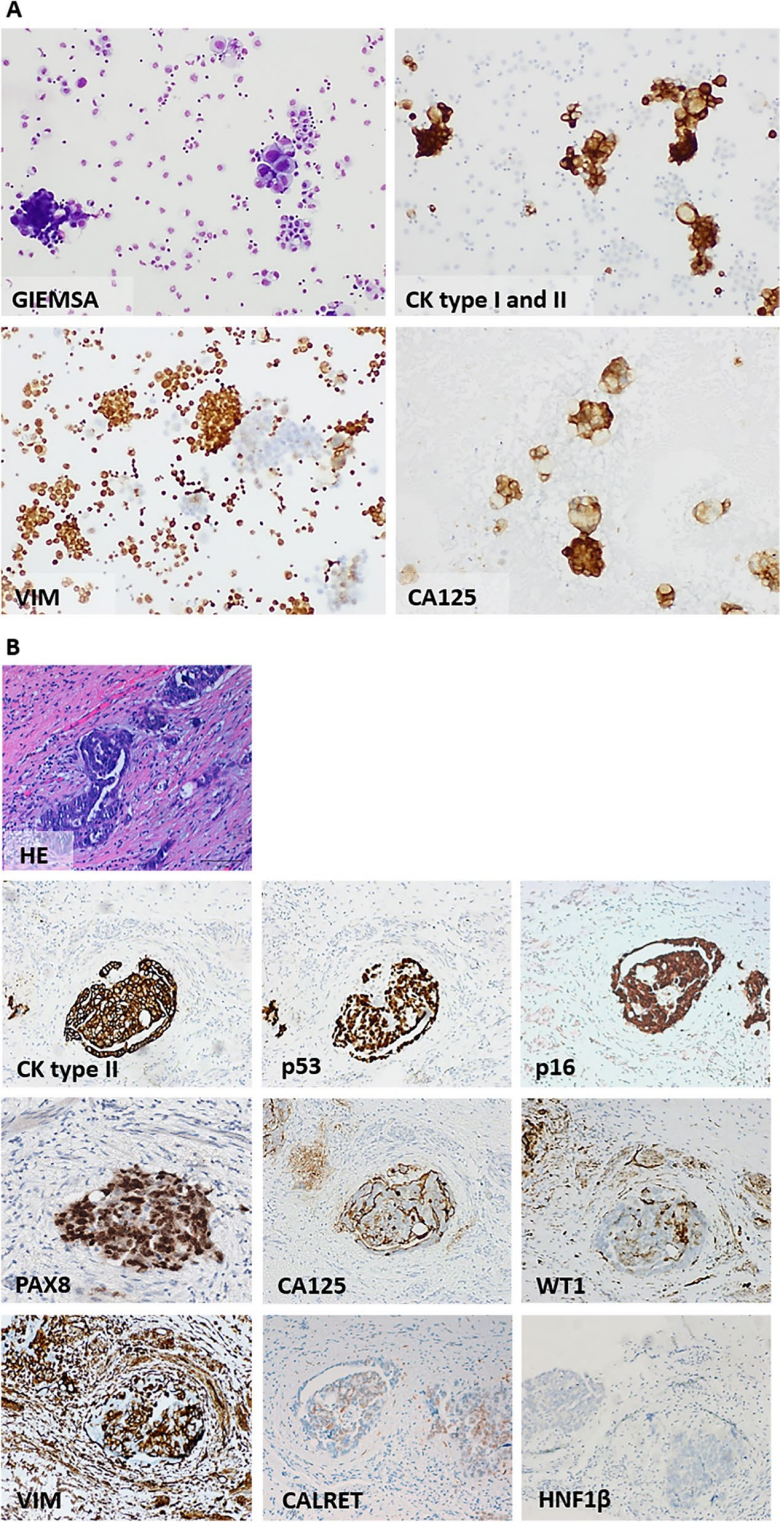
Morphological characterization of cells from ascitic fluid and characterization of the primary ovarian carcinoma of the patient from whose ascitic fluid IPO43 was established. **A** Cells were collected by cytospins. Smears of cells stained with Giemsa show aggregates and isolated cells with irregular size, abundant cytoplasm and large nuclei, confirming the presence of malignant cells; Immunocytochemical characterization of cells was done using cytokeratin (CK) type I and II—AE1/AE3 (neoplastic cells are positive); Vimentin (neoplastic cells are predominately negative, macrophages and mesothelial cells were positive) and CA125 (neoplastic cells were positive), 100x. **B** Formalin fixed paraffin-embedded sections of patient tumor ovarian high grade serous carcinoma submitted to surgery and neoadjuvant chemotherapy (after 3^rd^ cycle) stained with hematoxylin and eosin (H&E) and immunohistochemical staining. Tumor cells show positive expression of epithelial marker Cam 5.2 (CK type II). Apical cell membrane staining for CA 125 in tumor cells. Cells exhibited nuclear positive expression for p53, p16, PAX8 and focally with WT1. Tumor cells were negative with vimentin (VIM), calretinin (CALRET) and HNF1β, 100x

The ascitic fluid was observed in cytospins and cell morphology assessed by Giemsa staining (Fig. 1A). Malignant epithelial cells were present, in large cell aggregates or isolated cells surrounded by inflammatory and mesothelial cells, and expressed anti-cytokeratin type I and II (clone AE1/AE3) and anti-cancer antigen 125 (CA 125). Vimentin was seldom positive in few malignant cells (Fig. 1A).

### Establishment of the histological type of ovarian carcinoma

Residual tumor in the surgical specimen confirmed the diagnosis of HGSC and the immuno-profiling was done. Tumor cells were strongly positive with cytokeratin, CA 125, PAX8, p53 and p16 protein and were negative for Wilm’s tumor 1 protein (WT1), vimentin and anti-HNF1β (Fig. 1B). Some tumor cells expressed calretinin (CALRET).

### Expression of cell markers in IPO43 cell line

**Fig. 2:**
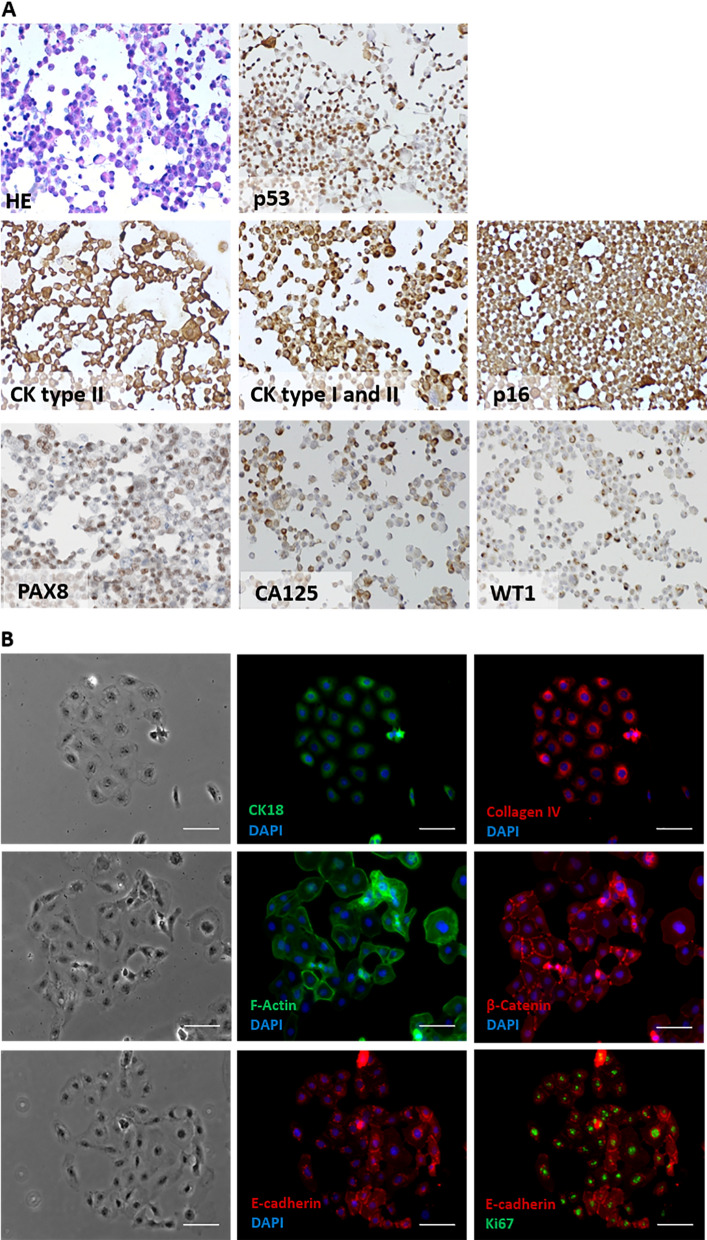
Expression of ovarian and biological markers in 2D cell culturing models in IPO43 cell line. **A** Cells were collected, prepared in cytospins and stained with hematoxylin and eosin (H&E) and several biomarkers were tested; cells show positive reaction with anti-cytokeratins (CK) type II (clone Cam 5.2) and type I and II (clone AE1/AE3). Cells exhibited protein expression for CA125, PAX8, p53 and p16. Cells were negative for WT1, 100x. **B** Representative morphological appearance of IPO43 cell line, phase contrast microscopy, 100x; cells exhibited positive expression for CK18, collagen IV, F-Actin, β-catenin, E-cadherin and Ki-67, 400x

The cell line expressed cytokeratins type I and II (clone Cam 5.2; AE1/AE3 cytokeratin 18) and exhibit staining for p53, WT1, p16^INK4a^, PAX8, CA 125, collagen IV, F-Actin, β-catenin and E-cadherin (Fig. 2A and B) and were negative for HNF1β (data not showed). A high proliferation evaluated by Ki-67 expression was found (around 70% of cells were positive).

### 2D morphological characterization of IPO43 cell line

**Fig. 3:**
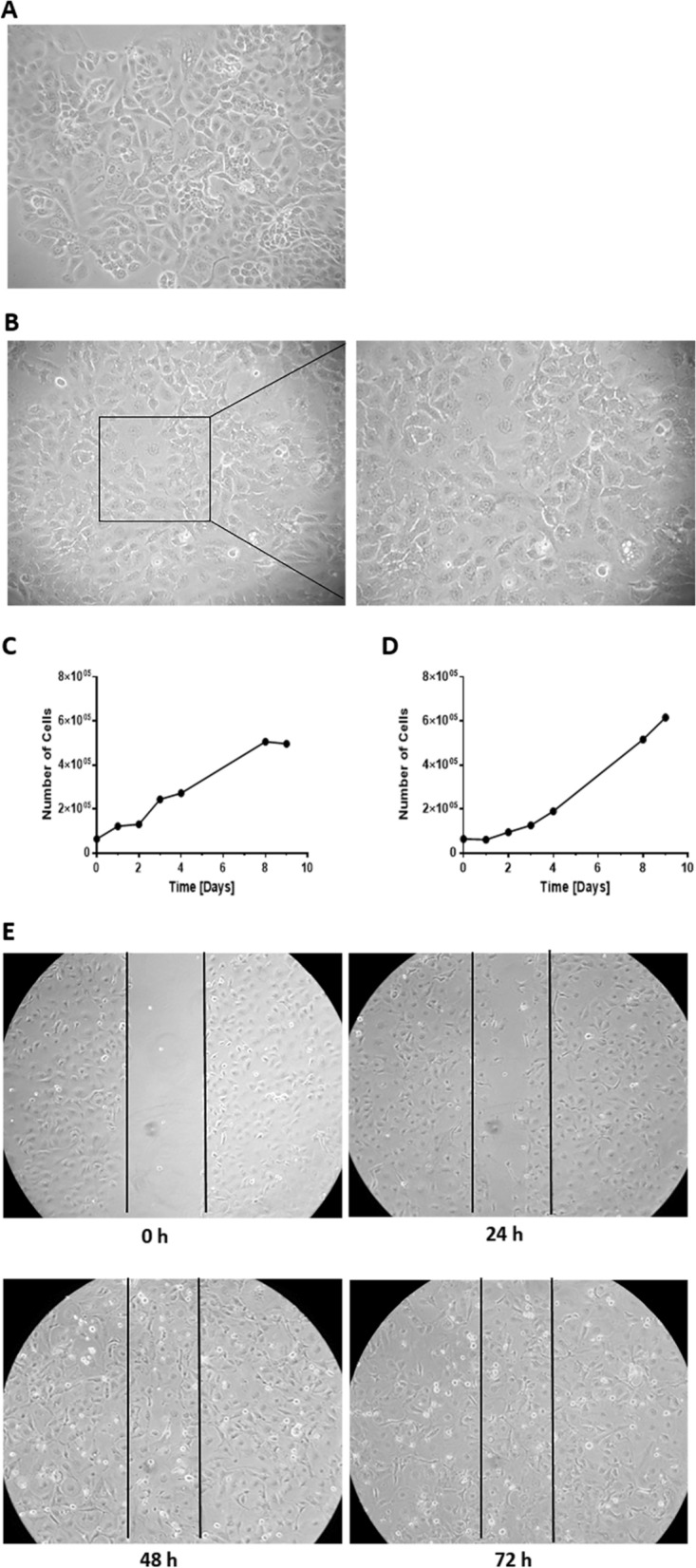
Characterization of IPO43 cell line in 2D model. The cell line presents cobblestone morphology or a pavement-like arrangement characteristic of epithelial cells in a confluent monolayer (cells with polygonal shape). **A** Representative morphological appearance of IPO43 cell line at passage 40, phase contrast microscopy, 100x; **B** Representative morphology of IPO43 cells at passage 80, phase contrast microscopy, 100x and 200x; **C/D:** Proliferation curve of IPO43 cell line, **C** Cell counting was determined by microscopy using the trypan blue exclusion method, and in **D** nuclei counting was determined by crystal violet staining, at regular time intervals (1 to 9 days) and **E** Wound healing assay to determine the migration rate of IPO43. Representative images of wound-healing assays performed in IPO43 cell. Cells were plated onto a 12 well dish and treated with mitomycin-C. Wound was generated and cells migrate filling the wound after 72 h. Pictures were taken at 0 h, 24 h, 48 h and 72 h after the scratch was performed (bright field microscopy), 100x

A monolayer of epithelial cells with a pavement-like arrangement was established (Fig. 3A—passage 40, 3B—passage 80). Cells were polygonal and showed atypical nuclear features such as clusters of chromatin, thickened and convoluted nuclear membrane, and large nucleoli. Some multinucleated giant cells were also seen in cell culture. The cells were able to adhere to the flask surface forming cell aggregates with polymorphic appearance.

**Fig. 4:**
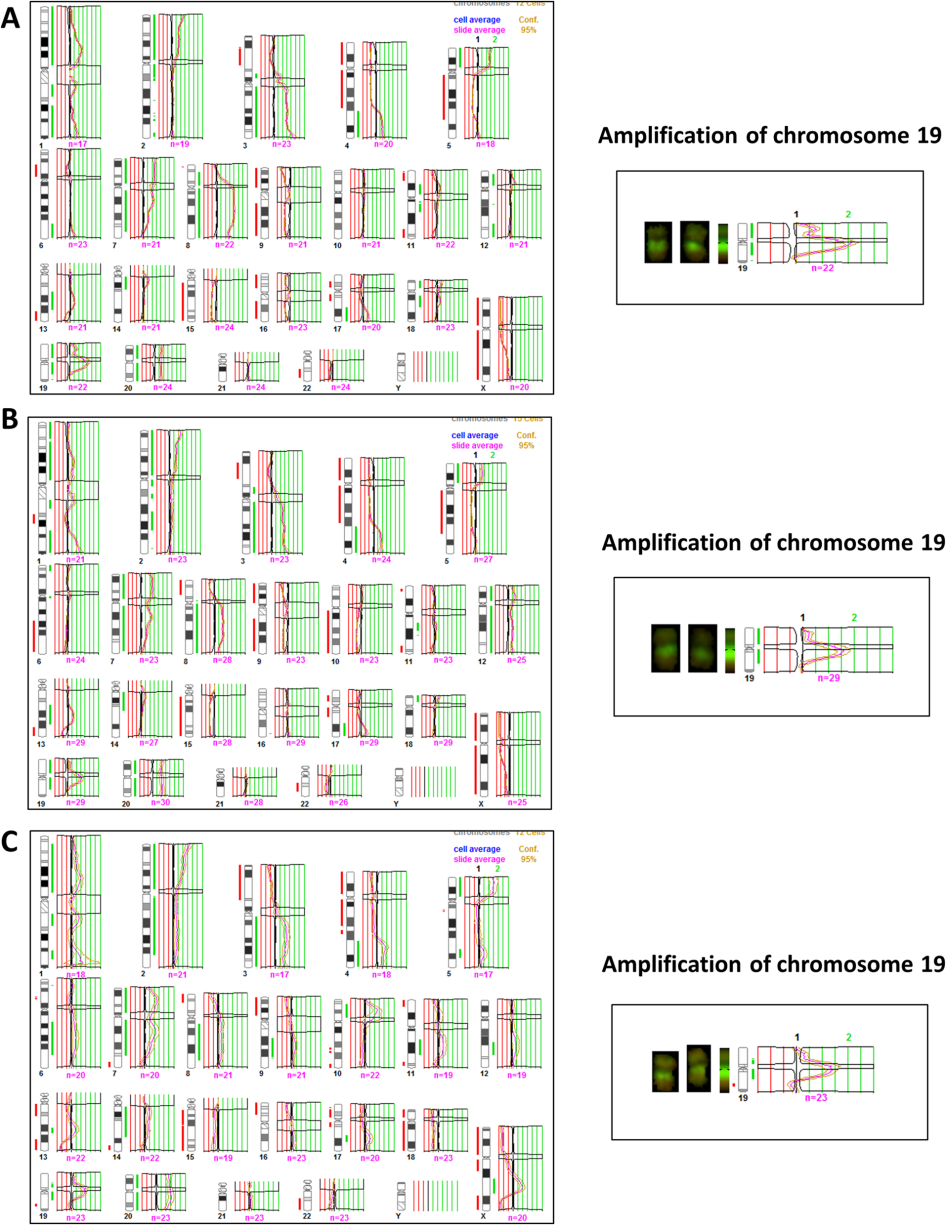
cCGH profiles of cells from ascitic fluid and IPO43 cell line.** A** Cells from ascitic fluid; **B** IPO43 cell line passage 20; **C** and passage 42; Red lines indicate loss and green lines indicate gain of chromosomal regions. Amplification of chromosome 19 is shown in the three passages.

These cells are growing without interruption for more than 50 months and with more than 100 serial passages, exhibiting a continuous, permanent and stable proliferation rate. This cell line is also maintained in DMEM with 1% of FBS (data not showed).

### Proliferation rate of IPO43 cell line

The proliferative characteristics of the cell line were assessed (Fig. 3C, D). The cell line exhibited a doubling time of 37.3 h, with a proliferation rate of 1.86% in the initial 72 h (3 days). Continuous growth (more than 100 passages) led us to believe that IPO43 cell line behaves as an immortalized cell line.

### Migration properties of IPO43 cell line

Cell migration measurement were done using the wound healing assay [12]. Scratch assays showed that the cell line have a low migration ability, taking 72 h to close the wound area (Fig. 3E).

### Clonal selection was observed in IPO43 cells by cCGH

The genomic profile by cCGH, analysis was performed from the original ascitic fluid cells (Fig. 4A) and in the corresponding cell line, at passage twenty (p20, Fig. 4B) and forty-two (p42, Fig. 4C) after its initial establishment. We also tried to perform cCGH analysis with DNA extracted from paraffin sections of the primary tumor but the quality of the DNA was insufficient.

As shown in Table 3 and Fig. 4, tumor cells from ascitic fluid showed a complex genomic profile by cCGH with several gains, including amplifications, and losses of chromosome regions. The cell line cCGH profile was quite similar exhibiting most of the alterations present in the original ascitic fluid but also showing some de novo alterations.

**Table 3:** Description of cCGH chromosome alterations in IPO43 cell line

	Gain of entire chromosomes/partial gains of chromosome regions	Loss of entire chromosomes/partial losses of chromosome regions
Cells from ascitic fluid	20/ 1p34.3-q23, 1q31-q32.1, 1q32.3-q44, p25-p11.2, 3p12-q29, 4q28-q35, 5p15.3-p13, 7p22-q34, 8p21-q24.3 with amplification 8q11.2-q23, 11p14-p11.2, 12p12-p11.2, 13q21-q31, 14q11.2-q13, 17q24-q25, 18p11.3-p11.2, 18q12, 19p13.3-q13.2 with amplification 19q12-q13.1	9, 15, 16, X/ 3p24-p14, 4p16-q28, 5q11.2-q31, 6p22-p12, 11p15, 11q22-q25, 13q32-q34, 17p13-q21, 18q21.3-q23, 19q13.3-q13.4, 22q11.2-q13
Cells passage twenty (p20)	7, 12, 20/ 1p36.3-q23, 1q32-q44, 2p25-q13, 2q21.1, 2q24-q32, 3p12-q29, 4q28-q35, 5p15.3-p13, 8q11.2-q24.3, 11q13-q14, 13q21-q31, 14q11.2-q21, 17q22-q25, 18p11.3, 19p13.3-q13.2 with amplification of 19q12-19q13.1	9, 15, X/ 1q25-q31, 3p23-p21, 4p16-q27, 5q11.2-q31.1, 6q21-q26, 8p23-p12, 10q11.2-q26, 11q23.3-q25, 13q32-q34, 17p12-q21.3, 22q12-q13
Cells passage forty-two (p42)	20/ 1p36.1-p31, 1q22-q23, 1q41-q42, 2p25-p21, 3q13.1-q29, 4q28-q35, 5p15.3-p13, 5q32, 7p22-q31, 8q13-q24.1, 9q22-q31, 10p13-p12, 14q11.2-q13, 17q22, 19p13.1-q13.1 with amplification of 19q12-19q13.1, Xq21.3-q25	15, 18/ 1q31.1, 3p26-p13, 4p16-q27, 5q11.2-q22, 6p21.3-p12, 7q35-q36, 8p23-p12, 9p24-p13, 17p13-q21.1, 19q13.3, 22q12-q13, Xp22.3-q21.3, Xq25-q28

The cells from ascitic fluid present alterations in TP53 gene, namely deletion of the region where TP53 gene is located, verified by cCGH (Fig. 4).

Other alterations, frequently associated in ovarian cancer and also reported in TGCA [13], such as 1p36.3, 8q11.2 and 14q11.2 were observed in IPO43 cell line. The acquisition of those changes that were not seen in the original ascitic fluid cells, possibly led to its spontaneous immortalization.

The cCGH analysis of the cells from passage twenty showed gains of chromosome 7, 12 and 20 and partial gains on several chromosomes, as reported in Table 3. The IPO43 cell line presents a total loss of chromosomes 9, 15 and X.

We also found amplification of chromosome 19 (19q12) that encodes Cyclin E gene, this feature is maintained throughout cell line passages and was reported in other studies [14, 15].

In the passage 42, IPO43 showed gain of chromosome 20 and partial gains in several chromosomes and again the amplification of 19q12-q13.1 (Fig. 4C). Loss of chromosome 15 and 18 was observed, at this passage, and the cell line also maintained partial losses of some chromosome regions. cCGH analysis revealed that IPO43 cells at passage 42 contained more genetic changes than the original tumor cells from ascitic fluid, however these data are consistent with the acquisition of genetic change with cell line passage and clonal evolution.

### Mutational status of *TP53*, *BRCA1* and *BRCA2* of IPO43 cell line

In order to further characterize the cell line, mutation analyses of *TP53* and *BRCA1* and BRCA*2* were performed. As mentioned above, IPO43 cell line has a deletion in the short arm of chromosome 17 in the region where TP53 is located, detected by cCGH, indicating the loss of one allele (Fig. 4). By automate Sanger sequencing a missense mutation in exon 5, codon 172 (p.V172F; GTT > TTT, c.514G > T) was detected in the other allele, with the substitution of Valin (Val) to phenylalanine (Phe), also identified in the hemizygotic histogram (data not showed) and by NGS. This variant c.514G > T, p. (Val172Phe) with an allelic fraction of 100% is classified in CinVar (Variation ID: 428,909) as probably pathogenic. Moreover, this mutation in TP53 is associated with an advantage for tumor growth in a variety of neoplasms and it might have a pathophysiological effect, as reported in V80 COSMIC database (http://cancer.sanger.ac.uk/cosmic/mutation) [16–21]. Thus, IPO43 cells do not have wild type p53 protein. This *TP53* mutation status of IPO43 is consistent with the intense nuclear positivity for p53 protein [22].

The analysis of mutations in *BRCA1* and *BRCA2* genes was performed by NGS. No pathogenic variants were identified in the *BRCA1* and *BRCA2* genes.

### STR profile of IPO43 cell line

**Fig. 5:**
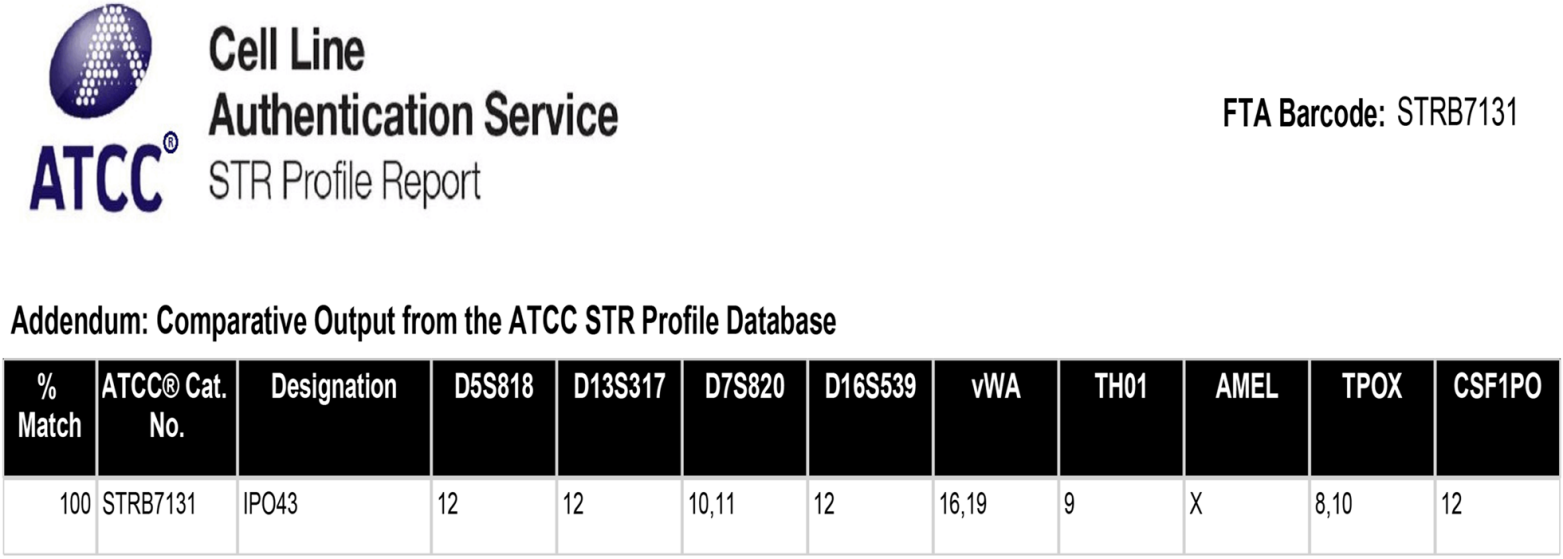
STR profile of IPO43 cell line. Cells were cultured on T-flaks, trypsinized and centrifuged at 125 × g. Cells were resuspended in PBS and cell density of 1 × 10^6^ cells/ml were spotted on FTA™ paper. STR profile of STR locis D5S818, D13S317, D7S820, D16S539, vWA, TH01, TPOX, CSF1PO and gender determination marker Amelogenin (AMEL)

**Fig. 6:**
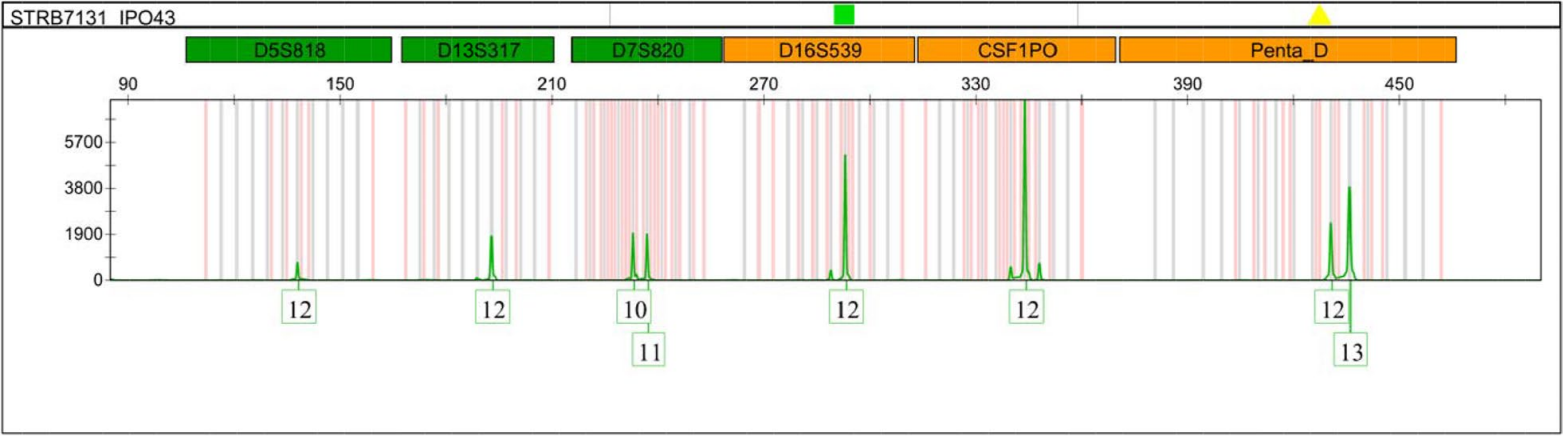
Electropherogram of IPO43 cell line. Electropherogram of STR locis D5S818, D13S317, D7S820, D16S539, CSF1PO and Penta D

In the present study, STR profile was analyzed by the PowerPlex® 18D Kit that amplifies 15 loci (D18S51, D21S11, TH01, D3S1358, FGA, TPOX, D8S1179, vWA, CSF1PO, D16S539, D7S820, D13S317, D5S818, D19S433, D2S1338), the gender specific marker Amelogenin (confirming that it is a female DNA) and two additional low-stutter and highly discriminating pentanucleotide STR loci, Penta E and Penda D (Figs. 5 and 6). It is evident that the mutations and karyotypic rearrangements presents in the cell line, highlighted by the cytogenetic analysis, confers to IPO43 cell line a molecular stability, also confirmed by the identification of the 17 loci generally used in human STR profiles.

So, STR profile confirms that the cell line profile is human, and is not a match to any cell line in either the ATCC or Expasy STR databases.

### Chemotherapy resistance of IPO43 cell line

**Fig. 7:**
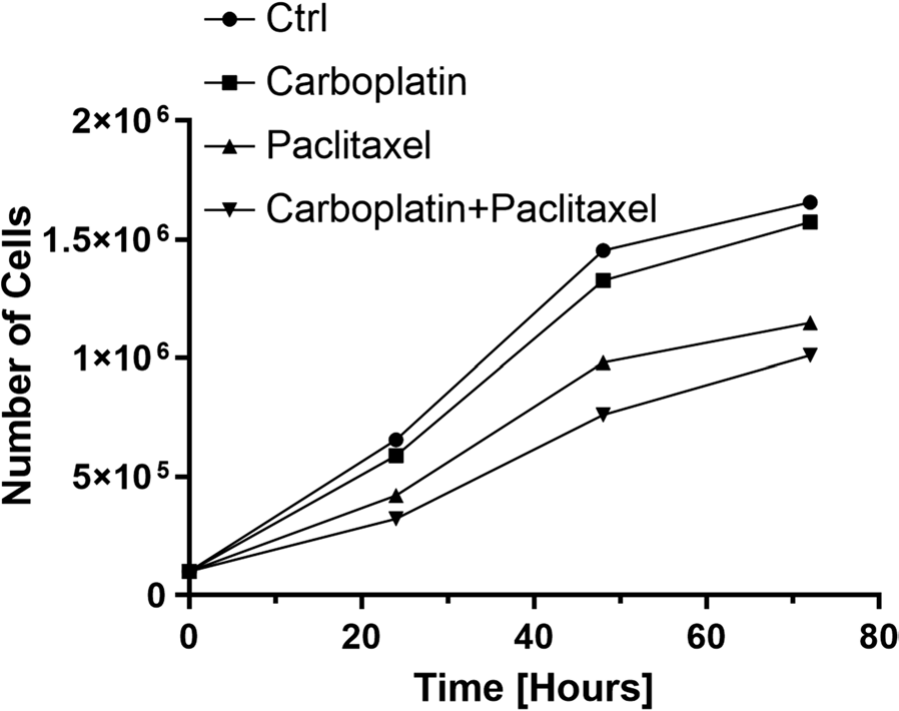
Growth curve of IPO43 cells during 72 h, after Carboplatin and Paclitaxel treatment. Cells were plated onto a 24 well plates and cell counting was determined by microscopy using the trypan blue exclusion method, and cultured in control condition and exposed to Carboplatin 25 μg/ml, Paclitaxel 10 μg/ml or both drugs during 72 h. Prior to any experiment, cells were synchronized under starvation (culture medium without FBS) for eight hours at 37 °C and 5% CO_2_

Chemotherapy resistance was evaluated by cell viability with proliferation curves. Using standard chemotherapy, there was no significant difference between the control and cell cultures with carboplatin addition. However, a slight decrease of proliferation was observed in cultured cells treated with paclitaxel alone or in combination with carboplatin (Fig. 7).

**Fig. 8:**
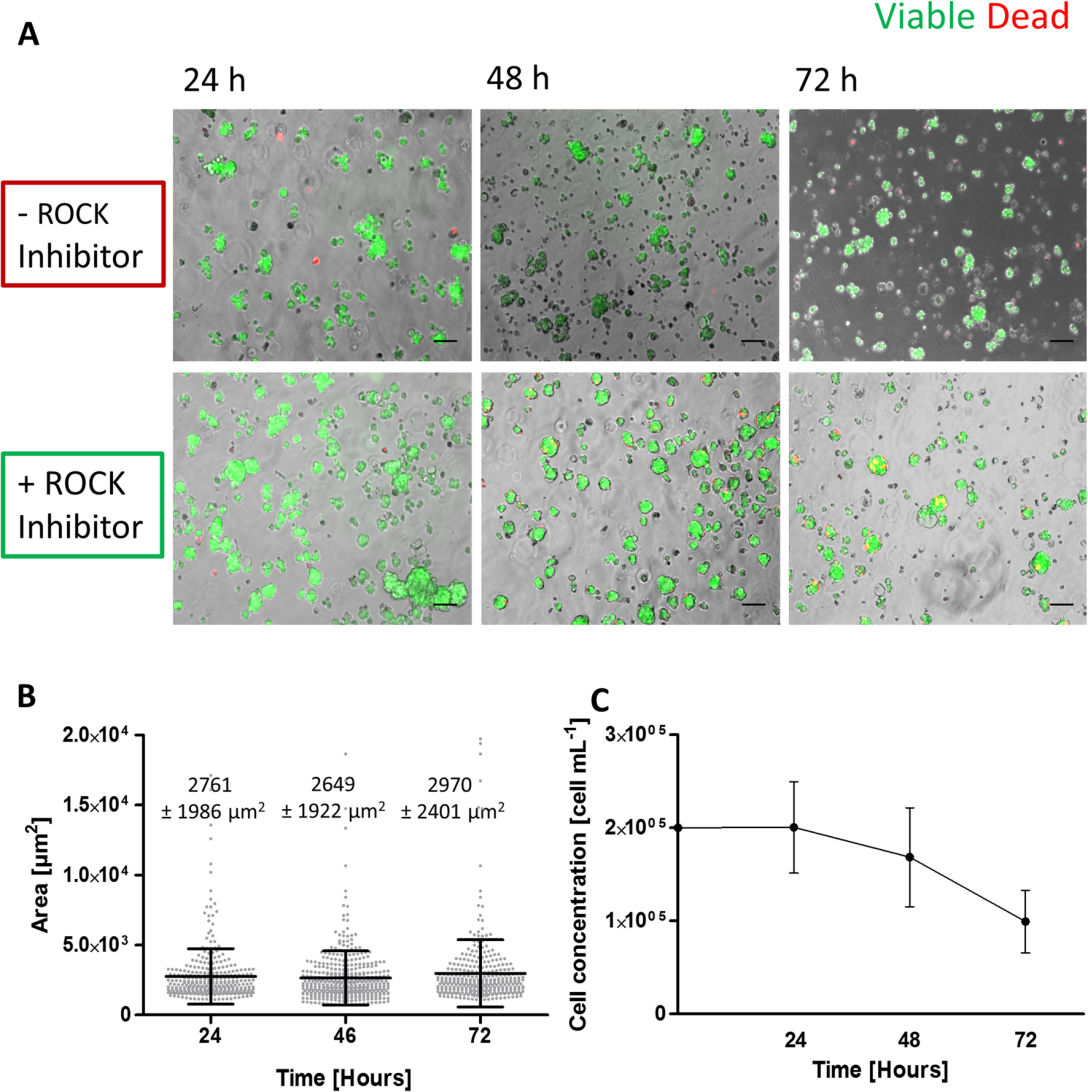
3D culture progression with measurement of aggregates area, growth curve and aggregation profile of IPO43 cells in the presence of ROCK Inhibitor in stirred-tank culture systems. **A** Aggregation of IPO43 with and without ROCK Inhibitor. Cells were cultured in a stirred-tank culture system placed on a magnetic stirrer (set at 40 rpm) and supplemented with and without ROCK Inhibitor, for 72 days. Supplementation with ROCK Inhibitor improved aggregation and after 72 h most aggregated cells are still viable, scale bar 100 µm. **B** IPO43 aggregates area and **C** Growth curve, determined by PicoGreen analysis. Values are presented as mean ± SD. Area increased with culture time, but growth tends to be lower

### Establishment and characteristics of 3D cell culture

3D cell cultures of IPO43 cells were established in stirred-tank culture systems. Two approaches were pursued to induce cell aggregation and compaction of cell aggregates: with and without ROCK inhibitor supplementation. We observed that IPO43 cells in 3D are heterogeneous and when cultured without ROCK inhibitor arranged in aggregates with irregular shape (grape-like shape, Fig. 8A). Addition of ROCK inhibitor improved cell aggregation reversibly as, when it was removed aggregates reacquired their original organization. After 72 h of culture the levels of cell death were lower in aggregates with ROCK inhibitor than without it (Fig. 8A).

Culture progression was evaluated by measuring the area of the tumor aggregates. Cell aggregates had an average area of approximately 2761 ± 1986 µm^2^ at day 1 of culture and 2970 ± 2401 µm^2^ at day 3 (Fig. 8B). We also assessed the cell concentration, by quantification of DNA and, after day 3, cell concentration decreased in 3D cultures (Fig. 8C).

These results show that IPO43 cells are suitable to establish 3D cultures and to generate cell spheroids.

**Fig. 9:**
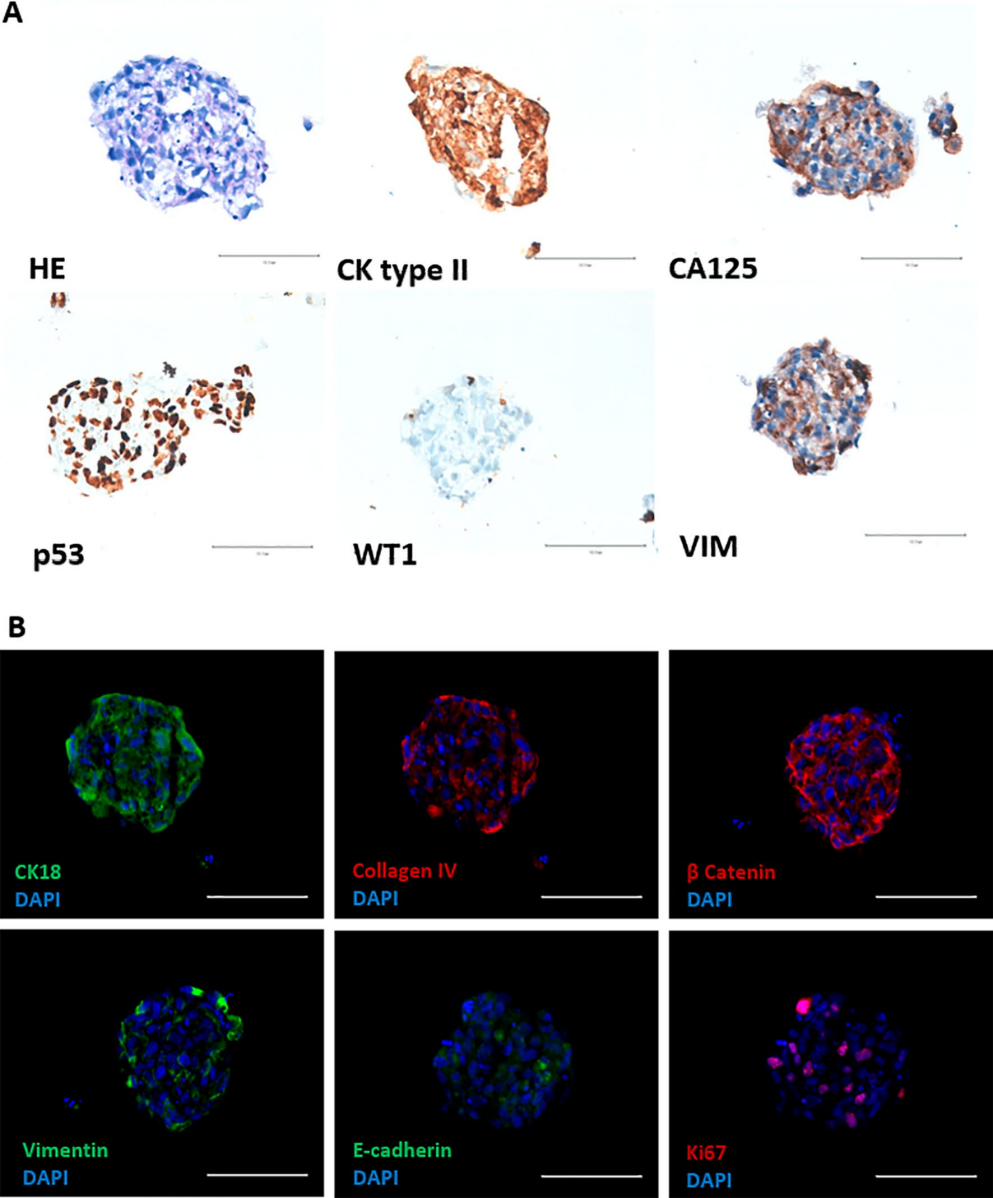
Characterization and expression of biological markers relevant for the establishment of 3D cell culturing models in IPO43 cell line. **A** Cryosections of 3D aggregates stained with hematoxylin and eosin (HE) and by immunohistochemistry. Cells show positive expression for cytokeratin (CK) type II (clone CAM 5.2), vimentin and p53. Cells were negative for WT1 and had positive expression for CA 125. **B** Cryosections of 3D aggregates show positive expression for CK18, collagen IV and β-catenin. Only few cells show positive expression for vimentin, E-cadherin and Ki-67, 400x

In 3D culture of IPO43 cells show the same biological markers (Fig. 9A) as in 2D (Fig. 2A) and exhibited loose aggregates. Cells in 3D continue to produce cytokeratin 18 and collagen IV; exhibited β-catenin, mainly in the cytoplasm, but fewer cells exhibited positive expression for E-cadherin, with a more peripheral distribution in cell aggregates. High proliferation activity is maintained as seen by Ki67 positive staining (Fig. 9B).

## Discussion

In this paper we characterized a new EOC cell line, IPO43, established in our laboratory that can be used as an important experimental tool in cancer research of EOC.

Cancer cell lines, besides their well-known limitations, are still useful in vitro models to study molecular mechanisms underlying cancer biology, chemoresistance and tumor recurrence, providing that they are well characterized [23]. Moreover, there are many benefits in using primary cell cultures with little or no laboratory-induced transformation as the behavior of these cells reflects better the original tumor cells.

Our cell line was established from a patient (73 years old) receiving neoadjuvant chemotherapy treatment for advanced HGSC, not manageable by surgery up front. Ovarian cancer is recognized as a disease with distinct molecular backgrounds [24] and not a single clinical entity. Seventy five percent of EOC are HGSC, making this tumor type an important research focus [25]. Although considerable progress has been made in cancer research, the survival rate of ovarian cancer has not improved over the past several decades [26], demonstrating the urgent need for understanding the mechanisms underlying the development of ovarian cancer.

In this study a detailed characterization of this cell line was done in order to establish the similarities and differences between the cell line and the original ascitic fluid [27].

The evaluation of the morphological features and immunohistochemistry confirmed that the IPO43 cell line resembles the original cells from the ascitic fluid cells and also from the patient’s ovarian primary tumor. The 2D model cell line expresses ovarian carcinoma markers and its epithelial origin has been confirmed by immunohistochemistry and immunofluorescence.

Dynamical chromosomal gains and losses in the cell line support the evidence of clonal selection. The cCGH profile of passage 42 of cell line showed less variability of numerical alterations apparently due to clonal adaptation to in vitro conditions and hopefully the stabilization of the cell line. Nevertheless, IPO43 cultured cells showed gains and losses similar to the parental cells as well as additional chromosomal alterations in clones compared with the parent cells suggesting that IPO43 cell line was undergoing clonal selection.

It is suggested that certain genetic alterations that emerge in a cancer cells confer a selective advantage over the others, and cancer progression results from the expansion of clone(s) that collectively acquires the following characteristics: self-sufficiency in growth signals, evasion to apoptosis, sustained angiogenesis, limitless replicate potential and capacity of tumor invasion and metastasis [28].

We verified that the cell line has a deletion of the region where TP53 is located and contains a missense mutation in *TP53* in exon 5, codon 172 (p.V172F;GTT > TTT, c.514G > T. Thus, IPO43 cells do not have wild type p53 protein that is an important marker of this tumor type.

IPO43 cell line also harbors gains in chromosome regions 19p13.1-19q13.2 with amplification at 19q12-19q13.1, that has been reported to be a frequent cytogenetic change in ovarian carcinomas by other studies [14, 29, 30]. The amplification of chromosome 19, where resides Cyclin E gene is the eight most common copy number amplified gene analyzed in all ovarian cancer samples included in The Cancer Genome Atlas (TCGA) [13] and has been identified as a primary oncogenic driver in a subset of high grade serous ovarian cancer that are resistant to chemotherapy [27, 31]. Beaufort et al. [3], describes other cell lines with similar characteristics to IPO43, such as OVCAR3, as being a cell line with epithelial morphology, originated from ascites with platinum based treatment, with TP53 mutation and amplification of Cyclin E, and also resistant to standard chemotherapy. Domcke et al. [5] also describe the same features for cell line COV318.

Other cytogenetic alterations that were already described as highly frequent in HGSC by Kim et al.[27], such as the gain of 1p36, Xq21 and loss of 8p23, gains of 5p15 and 14q11, and loss of 22q12, were also find in IPO43. We believe that our cell line may has become immortalized through these alterations as reported for other cell lines [14, 29, 30].

Finally, IPO43 cell line does not present *BRCA1* nor *BRCA2* mutations. These is similar to the previous two cell lines described [3].

In summary, the main genetic characteristics of this cell line match the “classical features” described by Cancer Genome Atlas Research Network (TGCA) [13] such as alterations in DNA regions copy number and *TP53* mutation (reported in 96% of cases) commonly associated to chemoresistant disease [31].

3D cultures of tumor cell lines are considered to be better models than 2D for evaluation of tumor characteristics. For this reason, we started by establishing a 3D culture of IPO43 and compared to the characteristics of neoplastic cells in the two culture types. IPO43 cells in 2D and 3D cultures expressed similar adhesion molecules such as E-cadherin and β-catenin; produce extracellular matrix macromolecules as collagen IV and cells preserved the phenotypical features of the original tumor. In both models, we observed a similar proliferation activity (evaluated by Ki-67).

The preservation of identical biological marker allows to generate an in vitro model and to explore the advantages of the use 3D cultures over the standard two-dimensional (2D) static culture [32, 33]; by reproducing better the effects of the tissue microenvironment, including cell morphology, polarity and junctions, providing better and more complete answers regarding neoplastic cells and their microenvironment [34].

## Conclusions

We describe the characteristic of an established cell line, IPO43, isolated from ascitic fluid of HGSC patient using different techniques and approaches (morphology, phenotype, genetic) and confirm its use as a stable cell line suitable for research on HGSC of ovary, fallopian tube and peritoneum.

## Abbreviations

EOC: Epithelial ovarian cancer
HGSC: High grade serous carcinoma
2D: Two-dimensional
FFPE: Formalin paraffin embedded tissue
CGH: Comparative genomic hybridization
HRP: Horseradish peroxidase
DAB: Diaminobenzidine
BSA: Bovine serum albumin
PBS: Phosphate buffered saline
PEG: Polyethyleneglycol
TBE: Tris/borate/EDTA
RT: Room temperature
FSG: Fish skin gelatin
DAPI: 4’,6-Diamidino-2-phenylindole
FITC: Fluorescein isothiocyanate
FDA: Fluorescein diacetate
EDTA: Ethylenediamine-tetraacetic acid
CK: Cytokeratin
HE: Hematoxylin–eosin
IHC: Immunohistochemistry
ROCK: Rho-associated coiled coil forming protein
